# Advanced intrahepatic cholangiocarcinoma treated using anlotinib and microwave ablation: A case report

**DOI:** 10.1097/MD.0000000000018435

**Published:** 2019-12-27

**Authors:** Aixia Zhang, Bin Liu, Dandan Xu, Yahong Sun

**Affiliations:** Department of Oncology, Affiliated Hospital of Shandong Academy of Medical Sciences, Jinan, China.

**Keywords:** advanced intrahepatic cholangiocarcinoma, anlotinib, microwave ablation, targeted therapy, tyrosine kinase inhibitor

## Abstract

**Introduction:**

Intrahepatic cholangiocarcinoma (ICC) accounts for 10% to 15% of all primary hepatic carcinomas. However, there are no effective drug treatment strategies against ICC, and surgery is currently the only curative treatment. Here, we present a case of ICC successfully treated with anlotinib, a novel oral agent.

**Patient concerns:**

The patient was a 66-year-old Han Chinese woman, and she was a retired worker. The patient had no history of hepatitis B infection or hypertension. Physical examination showed no abnormalities, and the patient showed no conscious discomfort. However, ultrasound revealed liver occupation.

**Diagnosis:**

Liver ultrasound and enhanced computed tomography (CT) indicated liver cancer with intrahepatic metastasis. Serum carbohydrate antigen 199 and alpha fetoprotein levels were high at 4270 and 1561 ng/mL, respectively. Pathologic findings of CT-guided liver biopsy revealed an adenocarcinoma. Owing to further immunohistochemical staining and clinical results, a diagnosis of ICC was made.

**Interventions:**

The patient had received 5 cycles of transhepatic arterial chemotherapy and embolization and 1 cycle of microwave ablation. Due to rapid tumor progression and loss of liver function, systemic chemotherapy was contraindicated. As second-line therapy, she received anlotinib, a novel tyrosine kinase inhibitor that inhibits tumor angiogenesis and proliferative signaling and has been used to treat refractory advanced non-small-cell lung cancer that shows progression despite treatment with ≥2 chemotherapy regimens.

**Outcomes:**

This patient showed a partial response after 2 cycles of treatment with anlotinib (12 mg daily, days 1–14 of a 21-day cycle). Drug-related side effects, such as hypertension and hand foot skin reaction, were observed. After 4 cycles of anlotinib, the efficacy appeared to be stable, and the patient showed a progression-free survival period of almost 4 months. However, the patient's condition worsened and she died of liver failure 6 months after treatment (overall survival, almost 6 months).

**Conclusion:**

Some cases of ICC may be responsive to the antiangiogenic drug, anlotinib, when combined with microwave ablation. Randomized clinical studies are required to further confirm the efficacy and safety of anlotinib in the clinical treatment of ICC.

## 1 Introduction

Intrahepatic cholangiocarcinoma (ICC) is a rare type of primary liver cancer that presents with non-specific clinical symptoms. ICC originates from the secondary branch of the intrahepatic bile ducts and the distal biliary tract epithelium.^[1]^ ICC is generally diagnosed at an advanced stage, and it has a high malignancy potential, poor prognosis, and a short median survival. The 5-year survival rate for ICC is 7%, while the 3-year survival rate among patients who do not undergo surgery is 0%.[^2^,^3^,^4^]

Currently, surgery is the only curative method for ICC. Several patients are not ideal candidates for radical surgical resection due to accompanying intrahepatic or distant metastasis at the time of diagnosis. Moreover, the postoperative recurrence rate in patients who undergo surgical treatment is approximately 60% to 90%.^[1]^ Currently the standard of care for patients with ICC who are not good surgical candidates is fractionated radiotherapy with or without chemotherapy.^[5]^ The current chemotherapy regimens for ICC mainly include gemcitabine, platinum, fluorouracil, and irinotecan. The median survival of patients with ICC who received gemcitabine + cisplatin chemotherapy was 11.7 months in one study.^[6]^ In addition to chemotherapy, microwave ablation (MWA) is widely used in the treatment of liver cancer due to its high thermal efficiency, fast heating, uniform high temperature heat field, complete necrosis in the area of coagulation, and minimal effect on blood flow.

Anlotinib (Zhengdatianqing Pharmaceutical Co., Ltd., LianYunGang, China) is a novel bioavailable small molecule tyrosine kinase inhibitor that inhibits several molecules involved in tumor progression, particularly vascular endothelial growth factor receptor type 2 and 3 (VEGFR-2,3), platelet-derived growth factor b (PDGFRb), and stem cell factor receptor (c-Kit). Anlotinib has been shown to confer progression-free survival (PFS) benefits for patients with refractory advanced non-small-cell lung cancer (RA-NSCLC) in phase III trials.^[7]^ In 2018, anlotinib was approved by the China State Food and Drug Administration for the treatment of RA-NSCLC in patients who had previously received ≥2 types of chemotherapy. Given that it targets multiple signaling pathways, anlotinib also has broad potential efficacy in the treatment of several solid tumors, including soft tissue sarcomas, medullary thyroid carcinoma, and metastatic renal cell carcinoma. Herein, we report a case of ICC in which anlotinib and MWA produced an excellent tumor response without serious treatment-associated side effects.

## 2 Case presentation

The patient was a 66-year-old Han Chinese woman, and she was a retired worker. The patient had no history of hepatitis B infection or hypertension. Physical examination showed no abnormalities, and the patient showed no conscious discomfort. However, ultrasound revealed liver occupation, and she was thus referred to a grade A hospital in Shandong province in July 2017. After admission to this hospital for additional testing, transhepatic arterial chemotherapy and embolization (TACE) was performed and the patient recovered well after surgery. Three additional cycles of TACE were then performed in this hospital in August, October, and December 2017. The patient recovered postoperatively without any complications.

**Figure 1:**
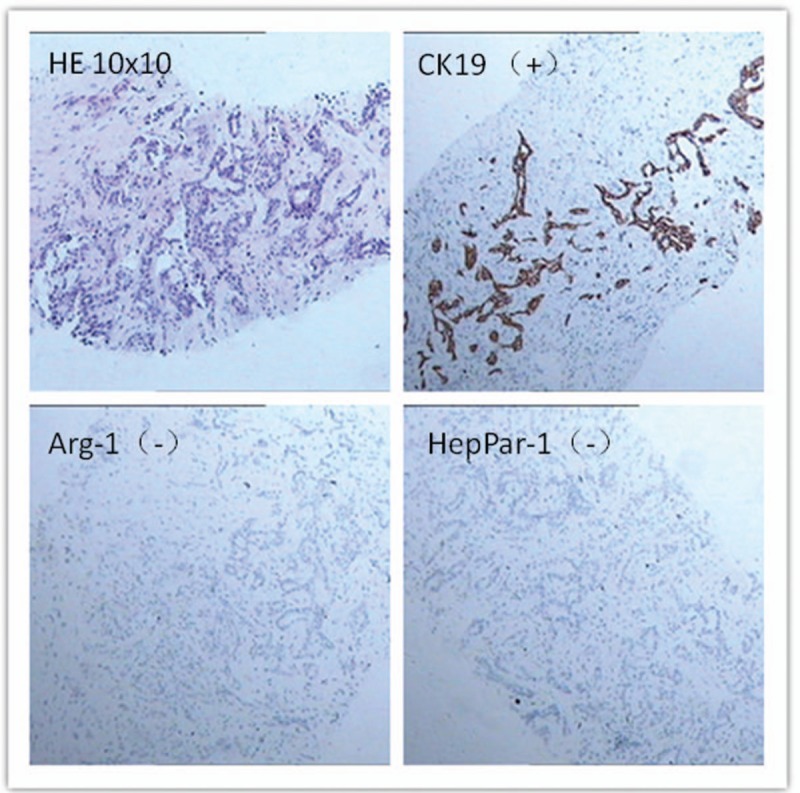
CA199 of the patients was 4270 ng/mL before treatment with antilotinib, 2371 ng/mL after one treatment cycle, and 575.6 ng/mL after 4 treatment cycles.

**Figure 2:**
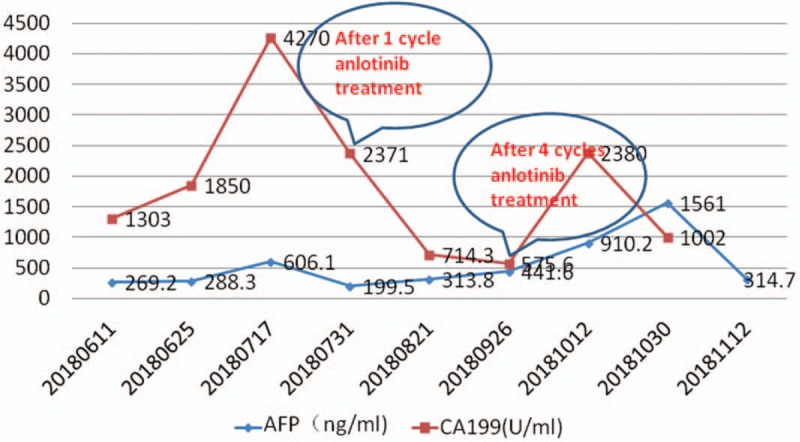
All indices of liver function of the patients improved to different degrees after 1 and 4 treatment cycles with anlotinib.

**Figure 3:**
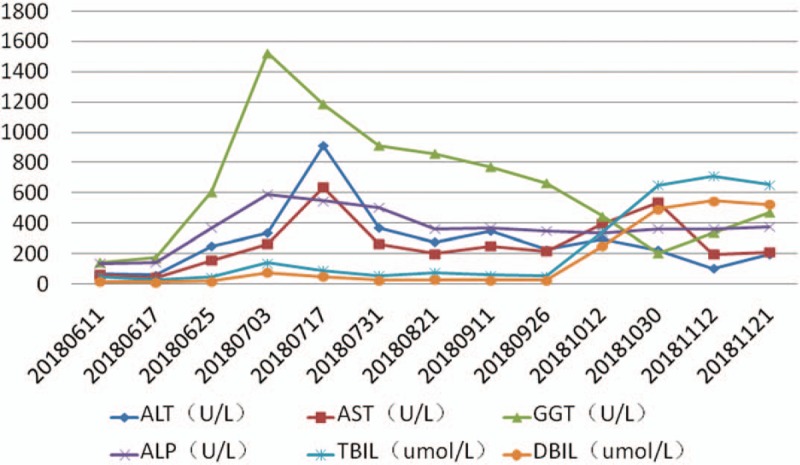
The patient was diagnosed with intrahepatic cholangiocarcinoma by liver biopsy and immunohistochemical analysis. (A) Pathological indication of adenocarcinoma (hematoxylin–eosin staining, ×100 magnification). Section showed positive staining for CK19 (B) and negative staining for Arg-1 (C) and HepPar-1 (D) (immunostaining, ×100 magnification).

On March 8, 2018 the patient was admitted to the oncology department of our hospital. Chest and abdomen computed tomography (CT) showed postoperative changes in liver cancer and intrahepatic metastases and multiple metastases in the lungs bilaterally. TACE was performed again on March 9, 2018, during which 50 mg of loplatin was injected, and recovery was achieved after surgery. On June 11, 2018, for the first time in our department, the patient showed no lack of consciousness and the Eastern Cooperative Oncology Group (ECOG) score was 0. A review of the enhanced chest and abdomen CT showed liver metastases and multiple metastases in the lungs bilaterally. The patient's alanine aminotransferase, aspartate aminotransferase, alkaline phosphatase, glutamyl transpeptidase, total bilirubin (TBIL), direct bilirubin (DBIL), alpha fetoprotein (AFP), and carbohydrate antigen 199 (CA-199) levels are shown in Figs. 1 and 2. CT-guided liver tumor biopsy and microwave ablation were performed on June 13. Postoperative pathological examination of the right hepatic neoplasm biopsy revealed an adenocarcinoma. Immunohistochemical staining results were as follows: wide CK(+); CK7(+); CK19(+); AAT(+); Villin(+); CA19-9(+); CK20(–); CDX2(–); HepPar-1(–); Arg-1(–); GPC-3(–); CEA(–); TTF-1(–); and Ki-67 (10%–15% [pathology no. 20180583]; Fig. 3). These results along with CT images led to a diagnosis of ICC. On June 25, 2018, liver function and AFP and CA-199 levels gradually increased (Figs. 1 and 2), and hepatoprotective therapy was performed. All indicators showed a continuous increase.

**Figure 4:**
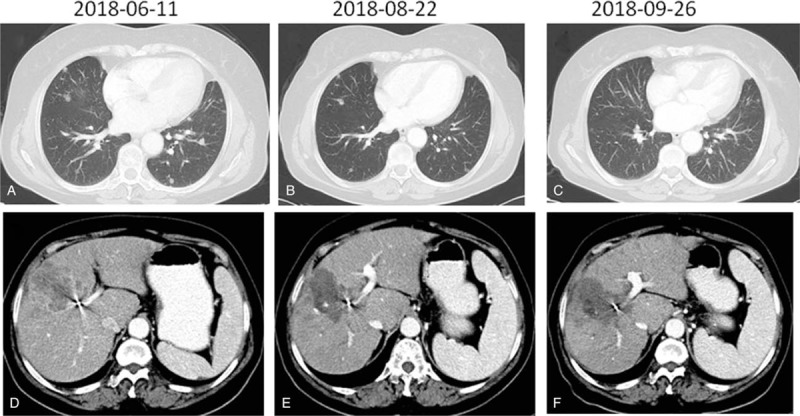
Computed tomography scan showed coexisting chest and abdominal masses (A and D) before treatment with anlotinib and microwave ablation. The sizes of the lesions in the chest and abdomen decreased significantly after combined treatment (B, C, E, and F).

During this time the patient presented with mild jaundice of the skin, oral mucosa, and sclera, as well as abdominal distension, weakness, and a decreased appetite. The ECOG score became 2 owing to tumor progression. Targeted therapy with anlotinib was started on July 11, 2018 at a dose of 12 mg once daily on days 1 to 14 of a 21-day cycle. After 4 cycles of anlotinib treatment, indices showed significant reduction (Figs. 1 and 2). The general condition of the patient improved, and the ECOG score became 1. After 2 cycles of anlotinib treatment, contrast-enhanced CT showed that both lung metastases and liver lesions had improved. Subsequent CT examination after 4 cycles revealed stable disease (Fig. 4). In early October 2018, the patient stopped taking anlotinib on her own, and showed yellow staining of skin mucosa and sclera. Her total bile acid level became 122.10 μmol/L, and TBIL, DBIL, and indirect bilirubin levels were 342.60, 249.39, and 93.21 μmol/L, respectively. We treated her with liver protection and deyellowing treatment and continued oral administration of anlotinib for 1 cycle. Re-examination showed that bilirubin levels continued to increase, and magnetic resonance cholangiopancreatography was performed in a hospital in Jinan city. The results showed that the junction between the left and right hepatic lobes was occupied, and the intrahepatic bile duct was dilated. Splenomegaly with a small amount of ascites was noted. On October 29, without any obvious trigger, the patient developed a large erythematous rash on the skin on the face, limbs, trunk, and oral mucosa. She experienced itching, cough, and chest tightness. After anti-allergy treatment, the patient showed improvement. On November 2, an ultrasound combined with digital subtraction angiography was performed for percutaneous hepatic puncture biliary drainage, and about 200 mL of bile was extracted every day after the operation. In the middle of November, slow reaction, obvious weakness, and aggravation of jaundice appeared. She was treated with hospice care and died of liver failure on December 4, 2018.

**Figure 5:**
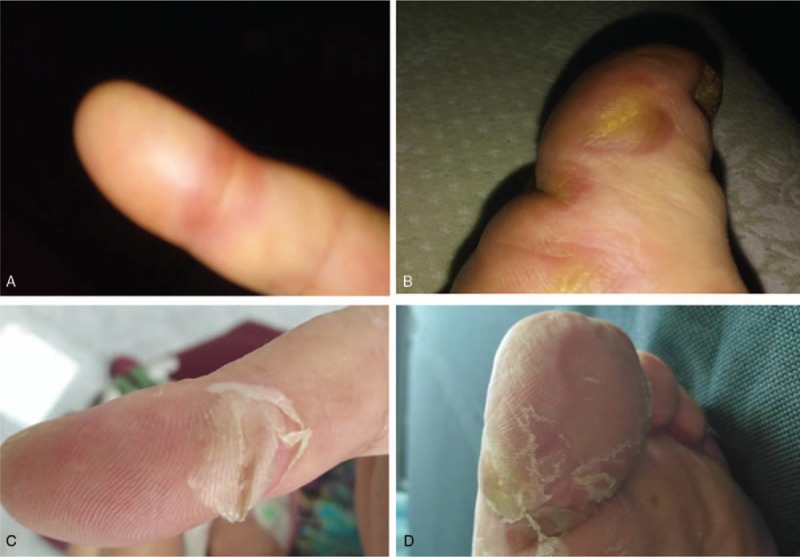
Manifestations of hand–foot syndrome after 2 cycles anlotinib treatment. (A, B) Performance after 2 treatment cycles. (C, D) Symptoms after 1 week of treatment withdrawal.

Treatment-related side effects were monitored weekly during anlotinib treatment. Hypertension (grade 3) and hand foot skin reaction (HFSR) (grade 3) were the main non-hematologic toxicities. HFSR (grade 3) occurred after 2 cycles of anlotinib treatment. The symptoms gradually decreased during the interval between cycles, and a selective COX-2 inhibitor (meloxicam) was administered to relieve pain while the patient was under treatment with anlotinib (Fig. 5). Hypertension was well-controlled by anti-hypertensive drugs. No other grade 3 to 4 side effects or other treatment-associated toxicities, such as proteinuria and hematologic toxicities, were observed. This patient did not have tumor lysis syndrome. She continues to receive anlotinib treatment and follow-up care.

## 3 Discussion

Approximately 395,000 new liver cancer cases are diagnosed in China each year, resulting in approximately 383,000 deaths. Liver cancer accounts for >50% of the global cancer incidence, and its incidence has been increasing by 5% each year.^[1]^ ICC accounts for 10% to 15% of all primary hepatic carcinomas. The incidence of ICC has shown an increasing trend in recent years among aging men. The cause of ICC is often related to chronic inflammation of the bile duct and physicochemical irritation. There is no effective drug treatment strategy for ICC. Surgery is the only curative treatment method for ICC, but many patients are not suitable candidates for radical surgical resection. In addition, although important in comprehensive treatment, radiotherapy, chemotherapy, interventional therapy, molecular targeted therapy, radiofrequency ablation, and seed implantation therapy have low treatment efficacy because of the poor differentiation of ICC.

MWA, a radical therapeutic method for liver cancer, is based on in situ inactivation of the tumor, while the surrounding liver tissue remains relatively normal.[^8^,^9^] MWA has the advantages of accurate positioning, simple and repeatable operation, reliability, less trauma to the body, low cost, and widespread indications. It has started to receive increased attention in clinical practice. MWA is suitable for single tumors with a diameter ≤5 cm or up to 3 small-to-medium-sized tumors with a maximum diameter of ≤3 cm. For large hepatocellular carcinomas with a diameter >7 cm, single-needle multi-point or multi-needle combined stacking technology can aid in complete tumor ablation. Liang et al^[10]^ reported the results of a multi-center study in which 1007 patients with primary liver cancer underwent MWA. The 1-, 3-, and 5-year survival rates were 91.2%, 72.5%, and 59.8%, respectively. The 5-year survival rate was similar to that for surgical resection and liver transplantation. Takami et al^[11]^ performed MWA on 719 patients with primary liver cancer and the 1-, 3-, 5-, 7-, and 10-year postoperative survival rates were 97.7%, 79.8%, 62.1%, 45.3%, and 34.1%, respectively. There was no statistical difference compared with other cases undergoing surgical resection at the same time. Although the complete ablation rate can reach up to 90%, high tumor recurrence rates remain a problem. Studies have shown that tumor number is an independent risk factor for early liver recurrence after liver cancer ablation (*P* < .05).[^12^,^13^]

Angiogenesis plays an important role in tumor growth, invasion, and metastasis.[^14^,^15^] Anlotinib is a new, small, orally administered multi-target tyrosine kinase inhibitor that was independently developed in China. Unlike other TKIs, anlotinib strongly inhibits multiple targets, such as VEGFR (VEGFR-1,VEGFR-2, and VEGFR-3), PDGFR (PDGFR-α and PDGFR-β), FGFR (FGFR-1, FGFR-2, FGFR-3, and FGFR-4), and c-kit.[^16^,^17^] For some kinases, such as Aurora B, c-FMS, and DDR1, various kinase mutants, such as Met and EGFR, have no apparent inhibitory activity. Anlotinib has an anti-tumor angiogenesis effect, inhibits tumor growth, and has apparent advantages in terms of the mechanism of action because its semi-inhibitory concentration (IC 50) for the above target is low and its safety is better. In a phase I study that evaluated anlotinib in patients with solid tumors, >60% of patients who received 12 mg once daily had tumor burden shrinkage.^[18]^ Han et al^[19]^ conducted the NCT01924195 trial to evaluate the safety and efficacy of anlotinib in drug-resistant NSCLC patients. A total of 117 patients were included in the study. Among the 117 patients, 60 received anlotinib (12 mg daily, orally; days 1–14 of a 21-day cycle). A placebo was administered to 57 patients at the same dose as that in the anlotinib group. The PFS of the anlotinib and placebo groups was 4.8 and 1.2 months, the ORR was 13.3% and 0%, and the DCR was 93.3% and 30%, respectively. ALTER-0303 is a Chinese multi-center randomized double-blind clinical study^[20]^ designed to evaluate the efficacy and safety of anlotinib as third-line treatment for advanced NSCLC. The main study endpoint was overall survival (OS). The results of the ALTER-0303 study were presented at the 2017 ASCO, and they showed significant improvements in OS (9.6 vs 6.3 months, *P* = .0018), PFS (5.4 vs 1.4 months, *P* < .0001), ORR (9.2% vs 0.7%, *P* < .0001), and DCR (81.0% vs 37.1%, *P* < .0001) in the anlotinib group compared with the control group. In this phase III study, there were no treatment-related deaths. The most common AEs were hypertension, elevated TSH levels, and HFSR. These AEs are similar to those of other TKIs.^[21]^

In this case, no histopathologic examination was performed at the time of initial diagnosis. TACE was performed multiple times, and no early surgical resection was performed. When admitted to our department, the primary lesion of liver cancer, intrahepatic metastasis, and bilateral lung metastasis were all significantly advanced. Liver tumor biopsy and MWA were performed. Because of the rapid tumor progression and liver function loss, the patient was not a candidate for systemic chemotherapy. Thus, we selected targeted therapy with anlotinib. The PFS was >3 months and OS was >5 months, which were good results. Adverse reactions such as hypertension and hand-foot syndrome were evident in patients, which could also be predictive indicators of response. The benefit might be better correlated with rapid tumor progression. In this setting, tumors with rapid progression are more likely to be dependent on new blood vessels and should have contained a higher percentage of immature, growth factor-dependent vessels.^[22]^ Similarly, in the ALTER-0303 study, all subgroup analyses showed that the anlotinib group had a significantly longer PFS in the >3 metastases subgroup.^[7]^

## 4 Conclusion

As a novel multitarget receptor tyrosine kinase inhibitor that can inhibit tumor angiogenesis and proliferative signaling, anlotinib has been approved by only the China State Food and Drug Administration as a third-line treatment for RA-NSCLC in patients who have received ≥2 types of chemotherapy. In this case report, anlotinib was shown to have good efficacy and safety for the treatment of a variety of solid tumors. It is possible that anlotinib is a feasible first-line treatment option for metastatic ICC. Large retrospective and prospective trials are needed to further confirm the efficacy and safety of anlotinib in the clinical treatment of ICC.

## Acknowledgments

This case report was approved by the Medical Ethics Committee of the General Hospital, Affiliated Hospital of Shandong Academy of Medical Sciences. Written informed consent was obtained from the patient for publication of this case report and accompanying images. They sincerely thank the patient for his contribution to the publication of this case report.

## Author contributions

**Data curation:** Aixia Zhang, Dandan Xu.

**Supervision:** Yahong Sun.

**Writing – original draft:** Aixia Zhang, Bin Liu.

**Writing – review & editing:** Aixia Zhang, Bin Liu, Dandan Xu, Yahong Sun.
